# Fluorescence-guided development of a tricistronic vector encoding bimodal optical and nuclear genetic reporters for *in vivo* cellular imaging

**DOI:** 10.1186/s13550-015-0097-z

**Published:** 2015-03-28

**Authors:** Adam Badar, Louise Kiru, Tammy L Kalber, Amit Jathoul, Karin Straathof, Erik Årstad, Mark F Lythgoe, Martin Pule

**Affiliations:** Division of Medicine, Centre for Advanced Biomedical Imaging (CABI), University College London, 72 Huntley Street, London, WC1E 6DD UK; UCL Cancer Institute, University College London, 72 Huntley Street, London, WC1E 6DD UK; Department of Chemistry and Institute of Nuclear Medicine, University College London, 235 Euston Road (T-5), London, NW1 2BU UK

**Keywords:** Reporter genes, SPECT, PET, BLI, Cell imaging, Multimodality imaging, Norepinephrine transporter, ASP^+^

## Abstract

**Background:**

*In vivo* imaging using genetic reporters is a central supporting tool in the development of cell and gene therapies affording us the ability to selectively track the therapeutic indefinitely. Previous studies have demonstrated the utility of the human norepinephrine transporter (hNET) as a positron emission tomography/single photon emission computed tomography (PET/SPECT) genetic reporter for *in vivo* cellular imaging. Here, our aim was to extend on this work and construct a tricistronic vector with dual optical (firefly luciferase) and nuclear (hNET) *in vivo* imaging and *ex vivo* histochemical capabilities. Guiding this development, we describe how a fluorescent substrate for hNET, 4-(4-(dimethylamino)styryl)-N-methylpyridinium (ASP^+^), can be used to optimise vector design and serve as an *in vitro* functional screen.

**Methods:**

Vectors were designed to co-express a bright red-shifted firefly luciferase (FLuc), hNET and a small marker gene RQR8. Genes were co-expressed using 2A peptide linkage, and vectors were transduced into a T cell line, SupT1. Two vectors were constructed with different gene orientations; FLuc.2A.RQR8.2A.hNET and hNET.2A.FLuc.2A.RQR8. hNET function was assessed using ASP^+^-guided flow cytometry. *In vivo* cellular conspicuity was confirmed using sequential bioluminescence imaging (BLI) and SPECT imaging of transduced SupT1 cells injected into the flanks of mice.

**Results:**

SupT1/FLuc.2A.RQR8.2A.hNET cells resulted in >4-fold higher ASP^+^ uptake compared to SupT1/hNET.2A.FLuc.2A.RQR8, suggesting that 2A orientation effected hNET function. SupT1/FLuc.2A.RQR8.2A.hNET cells were readily visualised with both BLI and SPECT, demonstrating high signal to noise at 24 h post ^123^I-meta-iodobenzylguanidine (MIBG) administration.

**Conclusions:**

In this study, a pre-clinical tricistronic vector with flow cytometry, BLI, SPECT and histochemical capabilities was constructed, which can be widely applied in cell tracking studies supporting the development of cell therapies. The study further demonstrates that hNET function in engineered cells can be assessed using ASP^+^-guided flow cytometry in place of costly radiosubstrate methodologies. This fluorogenic approach is unique to the hNET PET/SPECT reporter and may prove valuable when screening large numbers of cell lines or vector/mutant constructs.

**Electronic supplementary material:**

The online version of this article (doi:10.1186/s13550-015-0097-z) contains supplementary material, which is available to authorized users.

## Background

Cellular therapy with engineered cells is a promising area and is finding increasing application in regenerative medicine and cancer adoptive immunotherapy. In the clinical setting, T cell-based studies using chimeric antigen receptor (CAR) therapy have shown outstanding efficacy in refractory cancers [1-4]. More broadly, induced progenitor cells have been shown to regenerate different organs and systems in neurodegenerative [5], cardiovascular [6,7] and musculoskeletal [8] disorders. These therapeutics are different from standard therapies such as small molecules or proteins: they are essentially living therapies with no half-life, a complex engraftment and multifaceted *in vivo* behaviour.

A key limitation of pre-clinical and clinical development of advanced cellular therapies is lack of a satisfactory method for tracking these cells over sufficiently long periods of time. Direct or transient labelling methods, such as iron-labelled cells for magnetic resonance imaging (MRI) or ^111^In-oxine-labelled cells for single photon emission computed tomography (SPECT) are unsuitable for the time frame of immunotherapy studies. These allow imaging over hours/days [9-14], which are insufficient to study the complex biological integration and efficacy of cellular therapies, which can evolve over many weeks and months. A credible option is selective genetic modification of cells with a marker gene, using an integrating vector, to selectively track the cellular therapeutic indefinitely. Several different approaches to genetic reporter imaging have been proposed [15,16]. As a translational approach, positron emission tomography (PET) or SPECT show most promise. One such PET/SPECT genetic reporter is the human norepinephrine transporter (hNET) [17]. In combination with radiolabelled noradrenaline analogues, meta-iodobenzylguanidine (MIBG) or meta-hydroxyephedrine (mHED), hNET engineered cells can be mapped *in vivo* via SPECT (^123^I-MIBG) or PET (^124^I-MIBG, ^11^C-mHED) [17-19]. It has been demonstrated that the technique has quantitative capabilities and has been used in longitudinal imaging studies of adoptively transferred T cells and gene therapy [20,21].

Here our aim was to construct a tricistronic vector co-expressing hNET with firefly luciferase (FLuc) and a compact suicide/marker gene, RQR8 [22]. This triple construct extends upon the previously designed hNET-eGFP bicistronic construct [18]. The hNET-FLuc construct is a novel addition for *in vivo* bioluminescence imaging (BLI), and the triple construct of hNET-FLuc-RQR8 has potential for histochemistry and therapeutic application. In this study, several vector constructs were designed and tested. Screening for a lead construct, we show that hNET function can be assessed *in vitro* using ASP^+^, a fluorescent analogue of the neurotoxin MPP^+^ and hNET substrate [23,24], in place of conventional and costly radiosubstrate methodologies. Using this approach, we determined the optimal gene orientation of the tricistronic construct. Finally, we performed sequential BLI and ^123^I-MIBG SPECT *in vivo* imaging.

## Methods

### Generation of tricistronic retroviral vectors and transduction of SupT1 cells

All cell culture medium and supplements were obtained from Lonza BioWhittaker (Walkersville, USA) unless otherwise stated. A human T cell lymphoblastic lymphoma-derived cell line (SupT1) was obtained from the American Type Culture Collection (ATCC, University Boulevard Manassas, USA) and cultured in RPMI 1640 media (St. Louis, USA) supplemented with 10% foetal bovine serum (FBS) and GlutaMAX (Gibco, Grand Island, USA). Cell lines were maintained at 37°C in a humidified 5% CO_2_ atmosphere. Three SupT1 cell populations were engineered, each with a different expression cassette; (1) an IRES-based bisictronic vector encoding for hNET and a cell surface marker CD34 (vector 1: SupT1/hNET.I.CD34), (2) a 2A peptide-linked tricistronic vector encoding for hNET, red-shifted codon optimised FLuc [25] and a highly compact marker/suicide gene RQR8 [22] with 2A linkage at the N-terminus of hNET (vector 2: SupT1/FLuc.2A.RQR8.2A.hNET) and (3) at the C-terminus hNET (vector 3: SupT1/hNET.2A.FLuc.2A.RQR8). Moloney murine leukaemia virus (Mo-MuLV) long terminal repeat (LTR) promoter was used. SupT1 cells were transduced with RD114 pseudotyped supernatant generated from transfection of HEK-293T cells with the expression plasmids supplying Gagpol (gift from Elio Vanin, Baylor College of Medicine), RD114 envelope (gift from Mary Collins, University College London) PeqPam-env and each of the three hNET encoding vectors above. Transduction efficiency was determined by flow cytometry with αCD34-APC staining for vector 1 and QBEnd10 staining as previously described [22] for vectors 2 and 3.

### Optimisation of staining conditions for ASP^+^-guided flow cytometry

SupT1 cells (1 × 10^6^) transduced with hNET vector 1 were incubated at 37°C with varying concentrations of ASP^+^ (1 × 10^−3^, 5 × 10^−3^, 1 × 10^−2^, 5 × 10^−2^, 0.1, 0.5, 1 μM) for 10, 30, 60, 120 and 240 min. The cells were washed once with fluorescence activate cell sorting (FACS) buffer containing 1% FBS, phosphate saline buffer (PBS) and 50 mg/mL Normocin (InvivoGen, San Diego, USA). The supernatant was discarded and cells were re-suspended in 500 μL of FACS buffer. Flow cytometry was performed using Beckman Coulter Cyan instrument (Beckman Coulter, Brea, USA).

### ASP^+^-guided flow sorting and functional assessment of hNET expressing SupT1 cells

Optimal ASP^+^ staining conditions determined above were used for ASP^+^-guided flow sorting of SupT1 cell lines expressing the three hNET vector constructs. Cells were prepared as described above. ASP^+^ mean fluorescence intensities (MFI) were determined pre- and post-FACS (Beckman Coulter MoFlo-XDP) using areas under the curve and subtracting any non-specific signal from non-transduced SupT1 (SupT1/NT) cell populations. Measurements were acquired in triplicates for each cell line and statistical significance of differences between mean values was obtained with IBM SPSS software using the one-way ANOVA and the Tukey’s HSD test.

### *In vitro* radiotracer uptake assay

SupT1 cells expressing vectors 1 and 2 were further characterised by radiosubstrate uptake studies. 1 × 10^6^ SupT1/hNET.I.CD34, SupT1/FLuc.2A.RQR8.2A.hNET and SupT1/NT cells were incubated for 30 min at 37°C with [^125^I]-MIBG (7.4 kBq). The cells were rapidly washed twice with 500 μL ice-cold PBS and the supernatant collected. Cell pellets were resuspended in 1 mL PBS. Radioactive uptake was determined by gamma counting (WIZARD, PerkinElmer, Beaconsfield, UK) of the resuspended cells and corresponding supernatants. Percentage [^125^I]-MIBG cell uptake was calculated by dividing the counts in the cell pellet by the total counts (counts in cell pellet + counts in supernatant). All experiments were performed in triplicates. The one-way ANOVA and the Tukey’s HSD test were used to determine the significance of differences between mean values.

### *In vivo* bioluminescence and SPECT/CT imaging

All animal procedures were carried out in accordance with the UK Animals (Scientific Procedures) 1986 Act and institutional ethics regulation. SupT1/FLuc.2A.RQR8.2A.hNET cells (5 × 10^6^) or non-transduced SupT1 cells (5 × 10^6^) were injected into the subcutaneous space of the right flank of NOD/SCID mice (*n* = 3 in each group). Seven days later, BLI images (Biospace photon imager Optima system, Paris, France) were acquired 15 min post-intraperitoneal administration of 200 μL of D-Luciferin (10 mg/mL) using a 50 mm CCD lens with a sensitivity of 37 photons/s/sr/cm^2^. Images were analysed using Biospace M3 Vision software. 15 MBq (150 μL) of [^123^I]-MIBG (Mallinckrodt, Northampton, UK) was subsequently administered to animals via tail vein followed by whole body SPECT/CT imaging (nanoSPECT silver upgrade, Mediso, Budapest, Hungary) at 1 h, 4 h and 24 h post injection. Images were reconstructed using HiSPECT software (InviCRO, Boston, USA) followed by image processing and analysis using VivoQuant Software (InviCRO, Boston, USA).

## Results

### ASP^+^-guided flow cytometry enables functional assessment of hNET engineered cells

**Figure 1:**
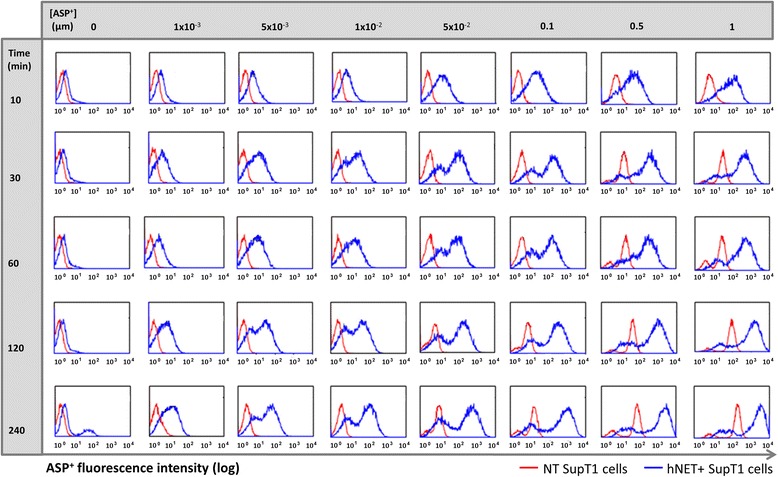
**ASP** ^**+**^ **staining optimisation of hNET expressing cells.** hNET positive (blue line) or non-transduced (red line) SupT1 cells were incubated with increasing [ASP^+^] from 0 to 1 μM. For each concentration, FACS was performed at 10, 30, 60, 120 and 240 min. Suggested optimal concentration and incubation time is 0.05 μM ASP^+^ and 30 min.

Functional determination of hNET vectors requires costly and complex radiochemistry work. We hypothesised that the fluorescent NET substrate ASP^+^ could allow determination of hNET function based on flow cytometry. A concentration and time-course titration assay was designed to determine optimised conditions for efficient cell staining and sorting of hNET expressing cells using ASP^+^. hNET expressing and non-transduced SupT1 cells were compared to quantify non-specific uptake (Figure 1). Adequate discrimination between specific and non-specific ASP^+^ staining is essential to study efficiency of hNET encoding vector transduction and reporter function and to facilitate isolation of pure hNET positive cell populations. We determined that ASP^+^ is both specifically and non-specifically taken up by cells (Figure 1). We observed good discrimination of specific and non-specific staining with a two-log shift in fluorescence. Optimal separation between hNET positive and hNET negative cells, using minimum incubation time and ASP^+^ concentration, was 30 min and 0.05 μM ASP^+^ (Figure 1).

### Tricistronic pre-clinical vector for histochemistry, BLI and SPECT tracking of engineered cells

**Figure 2:**
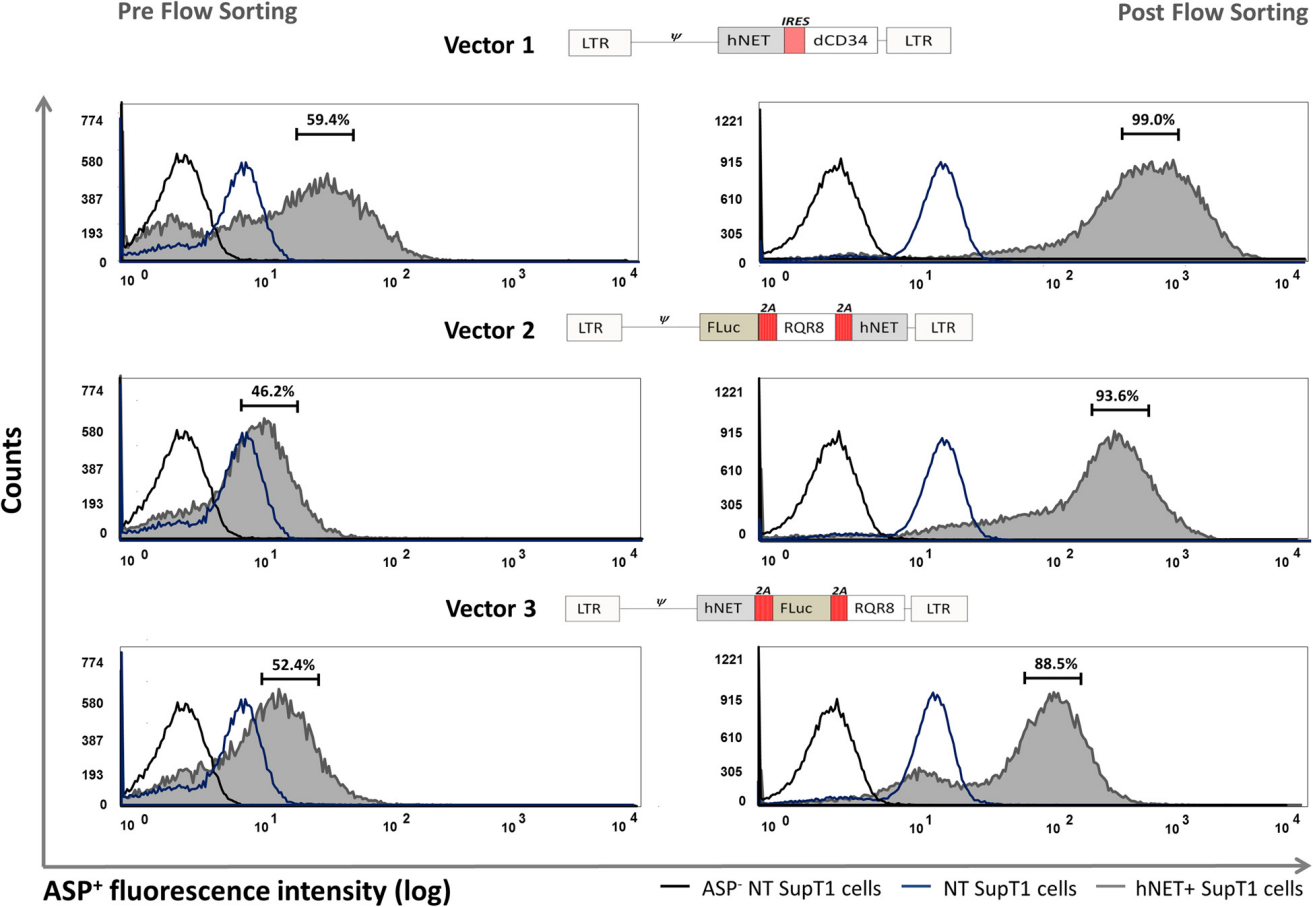
**Tricistronic hNET vector design and ASP** ^**+**^ **-guided FACS.** Three hNET encoding vectors were designed and transduced into SupT1 cells; hNET.l.dCD34 (vector 1), SupT1/FLuc.2A.RQR8.2A.hNET (vector 2), SupT1/hNET.2A.FLuc.2A.RQR8 (vector 3). Transduced (grey lines) and non-transduced (blue lines) cell populations were stained with ASP^+^ followed by flow cytometry (left column). ASP^+^-guided FACS was performed by gating on the brightest 5% within each of the three cell populations (right column).

We constructed a tricistronic vector which allows engineered cells to be tracked *in vivo* by BLI and SPECT/PET and *ex vivo* by histochemistry. We co-expressed a bright red-shifted firefly luciferase, along with the hNET and RQR8 - a small marker gene which binds the widely used antibody QBEnd/10 enabling flow cytometry and histochemistry. We used 2A peptide linkage to drive obligate stoichiometric co-expression [26]. Two candidate tricistronic vector constructs were designed and tested; one with 2A peptide linkage at the N-terminus of hNET (FLuc.2A.RQR8.2A.hNET) and one at the C-terminus (hNET.2A.FLuc.2A.RQR8). RQR8 function and transduction efficiency of the two vectors was verified via flow cytometry and QBEnd10 staining. As a reference, SupT1 cells encoding an IRES linked bicistronic vector (SupT1/hNET.I.CD34) were used. Using the optimal staining conditions determined above, ASP^+^-guided FACS was performed to isolate hNET positive SupT1 cells by gating on the brightest 5% of each population. Post sorting, positive ASP^+^ staining increased from 59.4% to 99% ± 0.01% for SupT1/hNET.l.dCD34, 46.2% to 93.6% ± 0.34% for SupT1/FLuc.2A.RQR8.2A.hNET, and 52.4% to 93.6% ± 0.66% for SupT1/hNET.2A.FLuc.2A.RQR8 (Figure 2). No difference in growth rates were observed in all cell lines compared to non-transduced SupT1 control cells. hNET function in all cell lines was tested over a period of 17 weeks via ASP^+^-guided FACS without any significant reduction in uptake observed (Additional file 1: Table S1).

### ASP^+^ screening to assess 2A peptide linkage orientation and hNET function

**Figure 3:**
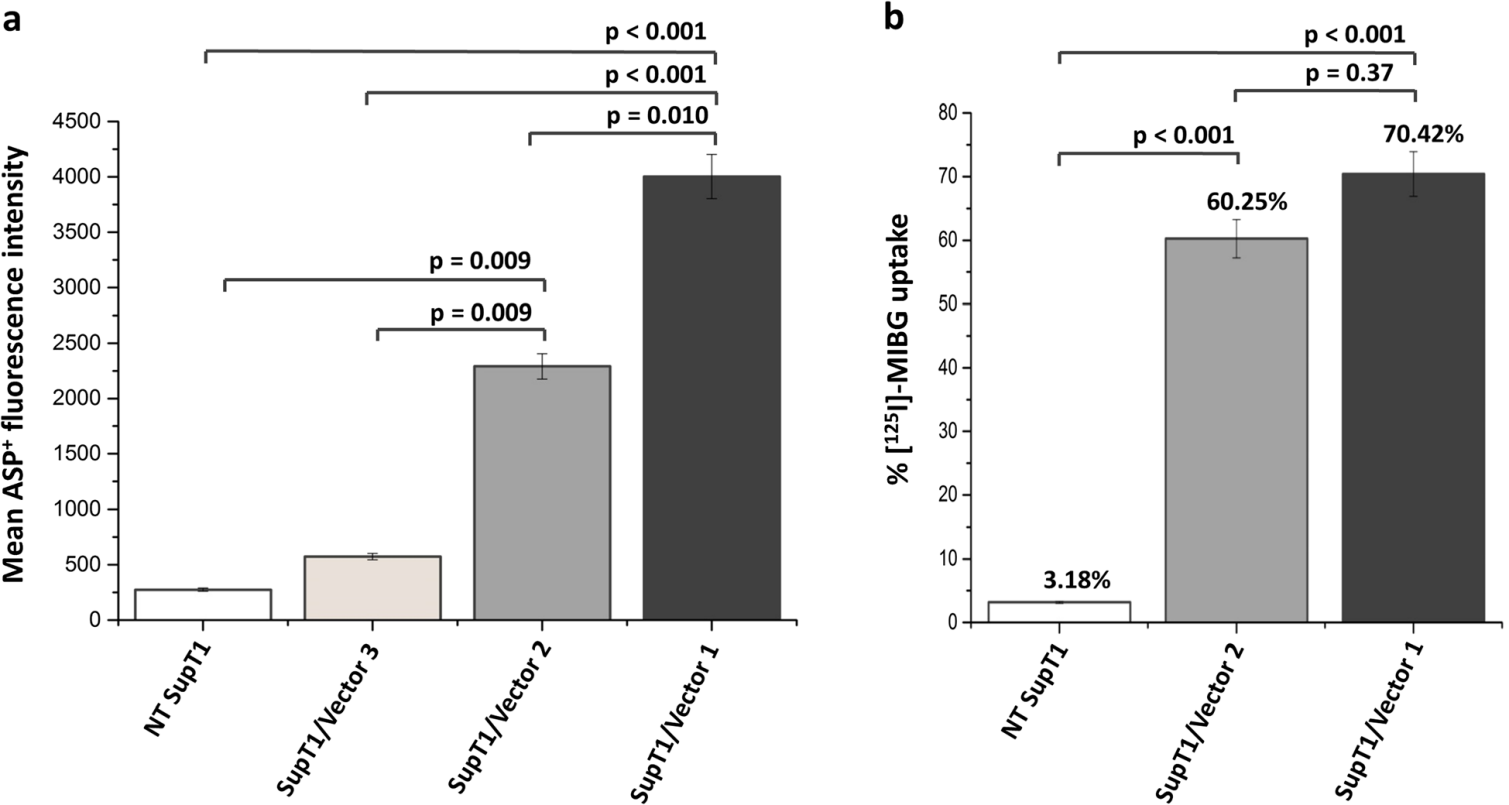
**Fluorescence and radionuclide cell uptake studies. (a)** ASP^+^-guided flow cytometry assessing hNET function in SupT1/NT (white), SupT1/FLuc.2A.RQR8.2A.hNET (beige), SupT1/hNET.2A.FLuc.2A.RQR8 (light grey) and SupT1/hNET.l.dCD34 (dark grey) sorted cells. Mean fluorescence intensities under the curves are presented. **(b)** hNET function in SupT1/hNET.2A.FLuc.2A.RQR8 cells was further characterised with an ^125^I-MIBG radiosubstrate uptake assay. Percent uptake was determined for SupT1/NT (white), SupT1/hNET.2A.FLuc.2A.RQR8 (light grey) and SupT1/hNET.l.dCD34 (dark grey) cells via gamma counting. Error bars are the mean ± SD for *n* = 3. Stats tests performed were ANOVA and Tukey’s HSD *post hoc*.

Interestingly, using ASP^+^, we found that 2A orientation within the tricistronic vector affected hNET function. Sorted SupT1/FLuc.2A.RQR8.2A.hNET and SupT1/hNET.2A.FLuc.2A.RQR8 cells were subject to ASP^+^-guided flow cytometry with mean fluorescence intensities measured as from the AUC. Despite resulting in a 1.7-fold lower MFI compared to the bicistronic reference cells (SupT1/hNET.l.dCD34), cells encoding the tricistronic vector with 2A at the N-terminus of hNET (SupT1/FLuc.2A.RQR8.2A.hNET) gave rise to a >4-fold higher MFI (2292.33 ± 80.39 MFI) compared to cells encoding 2A at the C-terminus of hNET (SupT1/hNET.2A.FLuc.2A.RQR8) (549 ± 20.43 MFI) (Figure 3a)*.* hNET function in SupT1/FLuc.2A.RQR8.2A.hNET cells was further characterised via radiosubstrate uptake assay. These cells demonstrated >18-fold higher ^125^I-MIBG uptake (60.25% ± 1.34%) compared to non-hNET expressing control cells (SupT1/NT) (3.18% ± 0.37%). Similar to ASP^+^ accumulation profiles, ^125^I-MIBG uptake in SupT1/FLuc.2A.RQR8.2A.hNET was 1.2-fold lower than in SupT1/hNET.l.dCD34 cells, suggesting reduced hNET pumping capabilities due to higher genetic load on tricistronic compared to bicistronic vector.

### *In vivo* imaging of the tricistronic vector

**Figure 4:**
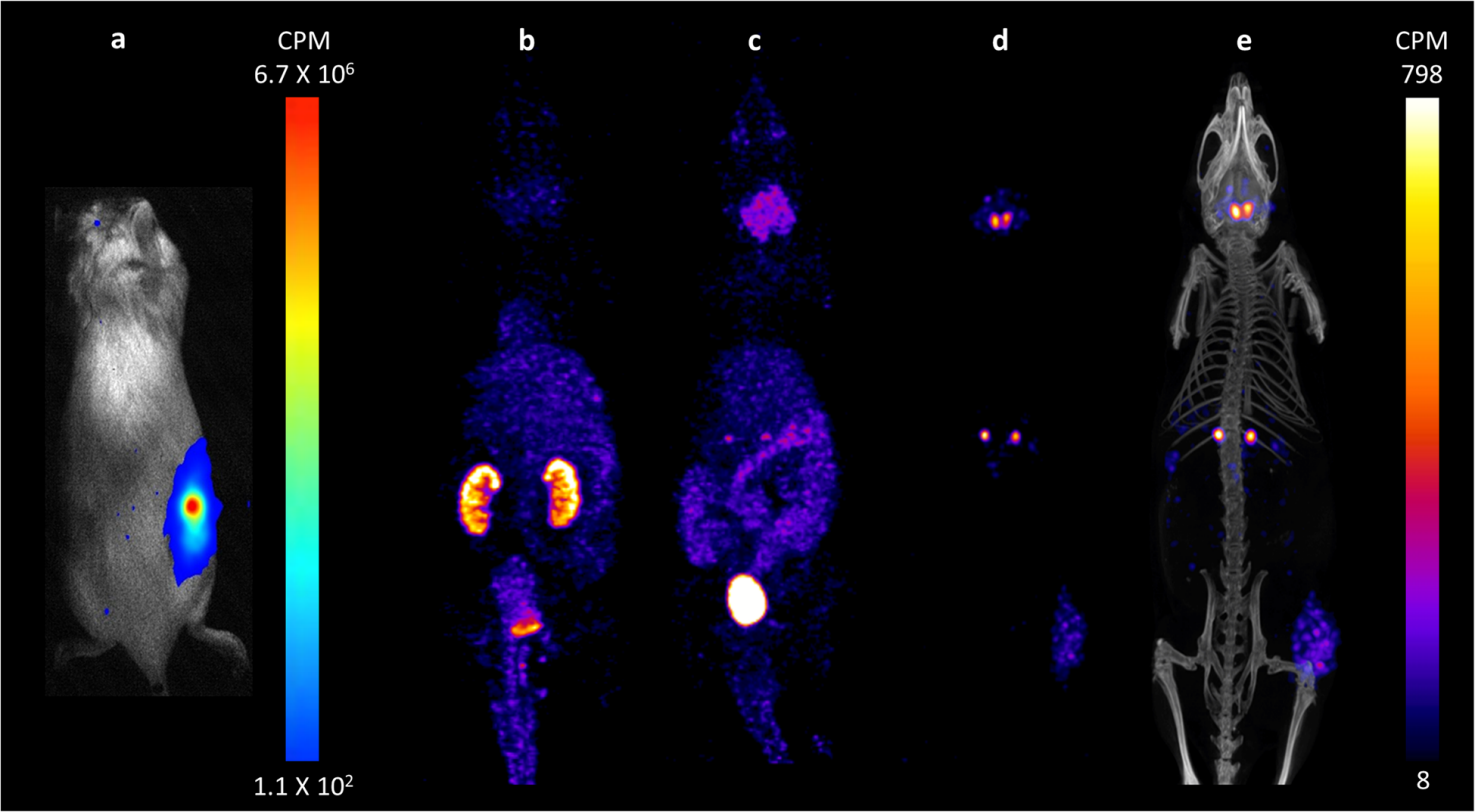
***In vivo*** **validation of the tricistronic vector hNET.2A.FLuc.2A.RQR8 via (a) BLI and (b-e) SPECT/CT.** 5 × 10^6^ SupT1/hNET.2A.FLuc.2A.RQR8 were inoculated into the right flank of immunocompromised mice. Regions of interest were drawn around the right flanks and mean luminescence signal intensity was measured (*n* = 3). **(b-d)** Maximum intensity projection (MIP) SPECT images and **(e)** SPECT/CT illustrate ^123^I-MIBG clearance via renal excretion, with increasing signal in the adrenals, thyroid, salivary glands and tumour **(d, e)**. Percent injected dose (*n* = 3) of ^123^I-MIBG within the tumours was determined by drawing 3D regions of interest. Colour bars represent counts per minute with maximum and minimum threshold levels indicated.

As an initial *in vivo* validation of the hNET encoding tricistronic bimodal imaging vector, 5 × 10^6^ SupT1/hNET.2A.FLuc.2A.RQR8 cells were inoculated into the flanks of immunocompromised mice and imaged 7 days later via BLI followed by SPECT/CT. Following luciferin administration, bioluminescence was clearly visible in the right flank (Figure 4a) (3.25 × 10^7^ ± 9.11 × 10^2^ CPM) indicating cell viability and FLuc function. Following intravenous tail vein injection of ^123^I-MIBG, dynamic whole body SPECT/CT images were collected at 30 min, 4 h and 24 h (Figure 4b,c,d,e). Consistent with earlier reports suggesting a late imaging paradigm is favourable when imaging hNET cells with MIBG [21], good signal-to-noise was observed at 24 h post radiotracer administration (Figure 4d,e). At the earlier time points of 30 min and 4 h, renal clearance is observed with high signal in the kidneys and bladder, with increasing signal in the adrenals (high NET expressing organ) (Figure 4b,c). Increasing signal was also observed in the salivary and thyroid glands (sodium iodide symporter expressing organs) due to tracer metabolism/deiodination (Figure 4b,c,d,e)*.* With decreased background signal at 24 h, radiotracer accumulation in the subcutaneous tumour can clearly be seen with regional heterogeneity resolved. Percent injected dose (% ID) of ^123^I-MIBG in tumours at 24 h post injection, as determined by drawing image-guided 3D regions of interest, was 3.12% ± 0.31% ID in hNET(+) xenografts, and 0.019% ± 0.01% ID in hNET(−) xenografts (equivalent to background levels).

## Discussion

With the development of new cellular and gene therapies, there is a need for non-invasive, readily available methods for detecting and quantifying the fate of these therapies in animal and man. As a translational approach, PET and SPECT genetic reporters have shown promise and have supported numerous clinical trials [27-29]. Various PET/SPECT reporter proteins have been proposed including the human sodium iodide symporter (hNIS), the hNET and the herpes simplex virus thymidine kinase (HSVtk) [16,17,30,31]. Difficulties in translating these into clinic include availability of clinically approved complementary radiotracers, early planning necessary to integrate the reporter protein and imaging protocol into trial design, incorporation of the reporter gene into the vector without exceeding the packaging capacity or affecting cell function and limited sensitivity in tissues with endogenous expression of the reporter protein.

In preclinical imaging, taking advantage of the strengths each modality (e.g. MRI, optical and nuclear) has to offer, the use of multimodal multicistronic reporters is becoming increasingly popular [16]. For example, co-expression of PET and BLI reporters would enable rapid, affordable and sensitive BLI, alongside complementary tomographic and quantitative PET capabilities. Expression vectors are often composed of multiple genes (e.g. a therapeutic, a marker gene, a suicide gene and a reporter gene) some of which can be large surface proteins, which may tax the vector packaging capacity and transcriptional efficiency. Strategies which promote maximising vector space are therefore essential and may include gene and protein truncation [22,32], efficient gene coupling approaches [33,34] and the utility of dual purpose genes [22,35,36].

Here, we have developed a trisictronic expression vector containing a nuclear reporter (hNET), a BLI reporter (FLuc) and a highly compact dual-purpose marker/suicide gene (RQR8). To achieve a compact vector, genes were linked with the ‘self-cleaving’ small 2A peptide, driving obligate co-expression under a single promoter (26). Exploiting the endogenous fluorescent properties of the hNET substrate ASP^+^, it was possible to simultaneously, and efficiently, sort pure cell populations and evaluate reporter function. Such a system is unique to hNET as a nuclear reporter and may circumvent the need for *in vitro* radioligand cell uptake assays prior to *in vivo* use. Furthermore, as hNET positive cells can be efficiently sorted using ASP^+^-guided flow cytometry, hNET could double up as a marker gene, leaving more flexibility to insert other genes in the expression vector.

Using ASP^+^-guided flow cytometry, we demonstrated that residual 2A amino acids attached to the C-terminus of the transcribed hNET markedly reduced the protein capacity to accumulate substrate into the cell. This finding further corroborates previous structure/function studies indicating the critical contribution of the hNET C-terminus to transporter trafficking, stability and function [37,38]. Consequently, an expression vector with 2A cleavage site upstream of hNET was taken forward for *in vivo* assessment. FLuc/hNET cells were successfully visualised using sequential BLI and SPECT imaging demonstrating high target to background tumour visualisation at 24 h post ^123^I-MIBG administration.

## Conclusions

In conclusion, exploiting the unique fluorescent properties of ASP^+^, we have been able to explore hNET function in engineered cells. We have constructed a novel pre-clinical tricistronic vector with BLI, SPECT, flow cytometry and histochemical capabilities, which can be widely applied in cell tracking studies supporting the development of cell therapies. Further investigation with regard to quantitatively correlating ASP^+^ and MIBG kinetics in hNET engineered cells, as well as determining the *in vivo* sensitivity of the technique is required and should be performed for each new cell line under investigation.

## Additional file

Additional file 1: Table S1. Functional assessment of hNET cell lines over time. SupT1 cells expressing hNET.l.dCD34 (vector 1), SupT1/FLuc.2A.RQR8.2A.hNET (vector 2) and SupT1/hNET.2A.FLuc.2A.RQR8 (vector 3) were incubated with ASP^+^ for 30 min at 37°C and the percentage of ASP^+^ cells was assessed using flow cytometry at weeks 1, 7, 11 and 17 post transduction.
