# Supplementary material for: Fluorescence-guided development of a tricistronic vector encoding bimodal optical and nuclear genetic reporters for in vivo cellular imaging
Source: EJNMMI Res. 2015 Mar 28;5:18. doi: 10.1186/s13550-015-0097-z (PMC4385325; doi:10.1186/s13550-015-0097-z)
Supplement: Additional file 1: Table S1. — Functional assessment of hNET cell lines over time. SupT1 cells expressing hNET.l.dCD34 (vector 1), SupT1/FLuc.2A.RQR8.2A.hNET (vector 2) and SupT1/hNET.2A.FLuc.2A.RQR8 (vector 3) were incubated with ASP+ for 30 min at 37°C and the percentage of ASP+ cells was assessed using flow cytometry at weeks 1, 7, 11 and 17 post transduction. [file 13550_2015_97_MOESM1_ESM.doc]

|  | **NT SupT1 (%)** | **Vector 1 (%)** | **Vector 2 (%)** | **Vector 3**  **(%)** |
| --- | --- | --- | --- | --- |
| **Week 1** | 1.02 | 94.20 | 99.07 | 98.08 |
| **Week 7** | 1.04 | 85.04 | 85.05 | 86.07 |
| **Week 11** | 0.99 | 86.81 | 85.00 | 85.07 |
| **Week 17** | 1.01 | 83.03 | 91.08 | 98.02 |

***Additional file 1: Table S1:*** *Functional assessment of hNET cell lines over time. SupT1 cells expressing hNET.l.dCD34 (vector 1), SupT1/FLuc.2A.RQR8.2A.hNET (vector 2) and SupT1/hNET.2A.FLuc.2A.RQR8 (vector 3) were incubated with ASP^+^ for 30 min. at 37°C and the percentage of ASP^+^ cells was assessed using flow cytometry at week 1, 7, 11 and 17 post transduction.*
